# Supplementary material for: Time-series transcriptome analysis identified differentially expressed genes in broiler chicken infected with mixed Eimeria species
Source: Front Genet. 2022 Aug 8;13:886781. doi: 10.3389/fgene.2022.886781 (PMC9393255; doi:10.3389/fgene.2022.886781)
Supplement: Supplementary file 2 [file DataSheet1.ZIP › 7dpi_KEGG.Gsea.1625115961950/KEGG_ADHERENS_JUNCTION.html]

Details for gene set KEGG\_ADHERENS\_JUNCTION[GSEA]

|  || Dataset | TMM\_7dpi\_gct\_format\_7dpi\_gct\_format.Class\_4dpi.cls #PC\_versus\_NC.Class\_4dpi.cls #PC\_versus\_NC\_repos |
| Phenotype | Class\_4dpi.cls#PC\_versus\_NC\_repos |
| Upregulated in class | 0 |
| GeneSet | KEGG\_ADHERENS\_JUNCTION |
| Enrichment Score (ES) | -0.48087883 |
| Normalized Enrichment Score (NES) | -1.6497716 |
| Nominal p-value | 0.0010960106 |
| FDR q-value | 0.067114405 |
| FWER p-Value | 0.4652 |
Table: GSEA Results Summary

  

Fig 1: Enrichment plot: KEGG\_ADHERENS\_JUNCTION      
 Profile of the Running ES Score & Positions of GeneSet Members on the Rank Ordered List

  

| SYMBOL | TITLE | RANK IN GENE LIST | RANK METRIC SCORE | RUNNING ES | CORE ENRICHMENT || 1 | BAIAP2 | na | 1322 | 0.424 | -0.0970 | No |
| 2 | ACP1 | na | 1501 | 0.381 | -0.0996 | No |
| 3 | CSNK2B | na | 1563 | 0.364 | -0.0929 | No |
| 4 | WASF1 | na | 1867 | 0.295 | -0.1088 | No |
| 5 | TCF7L1 | na | 1896 | 0.288 | -0.1018 | No |
| 6 | PTPRM | na | 2135 | 0.247 | -0.1138 | No |
| 7 | NLK | na | 2482 | 0.194 | -0.1365 | No |
| 8 | RAC1 | na | 2998 | 0.126 | -0.1755 | No |
| 9 | MET | na | 3037 | 0.122 | -0.1747 | No |
| 10 | WASF3 | na | 3124 | 0.111 | -0.1783 | No |
| 11 | ACTN1 | na | 3332 | 0.083 | -0.1930 | No |
| 12 | RHOA | na | 3449 | 0.068 | -0.2005 | No |
| 13 | SORBS1 | na | 3733 | 0.037 | -0.2230 | No |
| 14 | ERBB2 | na | 3936 | 0.011 | -0.2395 | No |
| 15 | FGFR1 | na | 4080 | -0.004 | -0.2514 | No |
| 16 | ACTB | na | 4316 | -0.032 | -0.2700 | No |
| 17 | ACTN4 | na | 4464 | -0.046 | -0.2808 | No |
| 18 | CREBBP | na | 5240 | -0.125 | -0.3417 | No |
| 19 | CSNK2A1 | na | 5588 | -0.157 | -0.3656 | No |
| 20 | WASL | na | 5673 | -0.167 | -0.3673 | No |
| 21 | CDC42 | na | 5837 | -0.184 | -0.3750 | No |
| 22 | RAC3 | na | 6076 | -0.210 | -0.3881 | No |
| 23 | PTPRF | na | 6124 | -0.217 | -0.3851 | No |
| 24 | TGFBR2 | na | 6133 | -0.218 | -0.3787 | No |
| 25 | CSNK2A2 | na | 6243 | -0.228 | -0.3805 | No |
| 26 | CTNNA1 | na | 6367 | -0.240 | -0.3830 | No |
| 27 | SRC | na | 6405 | -0.244 | -0.3782 | No |
| 28 | SNAI1 | na | 6637 | -0.269 | -0.3889 | No |
| 29 | CDH1 | na | 6672 | -0.272 | -0.3830 | No |
| 30 | TCF7L2 | na | 7019 | -0.311 | -0.4019 | No |
| 31 | TCF7 | na | 7360 | -0.347 | -0.4191 | No |
| 32 | TGFBR1 | na | 7506 | -0.362 | -0.4196 | No |
| 33 | EP300 | na | 7828 | -0.398 | -0.4336 | No |
| 34 | MAP3K7 | na | 7867 | -0.403 | -0.4238 | No |
| 35 | RAC2 | na | 7904 | -0.409 | -0.4136 | No |
| 36 | CTNND1 | na | 7979 | -0.417 | -0.4063 | No |
| 37 | CTNNA3 | na | 8161 | -0.441 | -0.4072 | No |
| 38 | PARD3 | na | 9042 | -0.554 | -0.4630 | Yes |
| 39 | CTNNB1 | na | 9201 | -0.583 | -0.4574 | Yes |
| 40 | SSX2IP | na | 9336 | -0.604 | -0.4491 | Yes |
| 41 | WASF2 | na | 9466 | -0.626 | -0.4397 | Yes |
| 42 | NECTIN1 | na | 9613 | -0.648 | -0.4310 | Yes |
| 43 | LMO7 | na | 9665 | -0.656 | -0.4141 | Yes |
| 44 | YES1 | na | 9689 | -0.659 | -0.3948 | Yes |
| 45 | PTPN6 | na | 9763 | -0.668 | -0.3793 | Yes |
| 46 | IGF1R | na | 9854 | -0.683 | -0.3648 | Yes |
| 47 | SMAD3 | na | 10010 | -0.714 | -0.3548 | Yes |
| 48 | TJP1 | na | 10016 | -0.715 | -0.3321 | Yes |
| 49 | MAPK3 | na | 10320 | -0.774 | -0.3325 | Yes |
| 50 | VCL | na | 10395 | -0.790 | -0.3132 | Yes |
| 51 | CTNNA2 | na | 10671 | -0.847 | -0.3089 | Yes |
| 52 | NECTIN3 | na | 11121 | -0.970 | -0.3151 | Yes |
| 53 | FARP2 | na | 11195 | -0.999 | -0.2890 | Yes |
| 54 | PTPRJ | na | 11293 | -1.033 | -0.2638 | Yes |
| 55 | SNAI2 | na | 11384 | -1.073 | -0.2367 | Yes |
| 56 | LEF1 | na | 11388 | -1.076 | -0.2022 | Yes |
| 57 | FYN | na | 11389 | -1.076 | -0.1675 | Yes |
| 58 | EGFR | na | 11415 | -1.085 | -0.1346 | Yes |
| 59 | FER | na | 11437 | -1.094 | -0.1010 | Yes |
| 60 | INSR | na | 11461 | -1.101 | -0.0674 | Yes |
| 61 | PTPN1 | na | 11465 | -1.103 | -0.0320 | Yes |
| 62 | AFDN | na | 11645 | -1.199 | -0.0083 | Yes |
| 63 | PTPRB | na | 11649 | -1.201 | 0.0302 | Yes |
Table: GSEA details [plain text format]

  

Fig 2: KEGG\_ADHERENS\_JUNCTION      
 Blue-Pink O' Gram in the Space of the Analyzed GeneSet

  

Fig 3: KEGG\_ADHERENS\_JUNCTION: Random ES distribution      
 Gene set null distribution of ES for **KEGG\_ADHERENS\_JUNCTION**
